# Isolation and Characterization of the Progenitor Cells From the Blastema Tissue Formed at Experimentally-Created Rabbit Ear Hole

**Published:** 2013-02

**Authors:** Mohamadreza Baghaban Eslaminejad, Sima Bordbar

**Affiliations:** 1 Department of Stem Cells and Developmental Biology, Cell Science Research Centre, Royan Institute for Stem Cell Biology and Technology, ACECR, Tehran, Iran

**Keywords:** Blastema, Differentiation, Proliferation, Rabbit

## Abstract

***Objective(s)*** *: *Throughout evolution, mammalians have increasingly lost their ability to regenerate structures however rabbits are exceptional since they develop a blastema in their ear wound for regeneration purposes. Blastema consists of a group of undifferentiated cells capable of dividing and differentiating into the ear tissue. The objective of the present study is to isolate, culture expand, and characterize blastema progenitor cells in terms of their* in vitro* differentiation capacity.

***Materials and Methods:*** Five New Zealand white male rabbits were used in the present study. Using a punching apparatus, a 4-mm hole was created in the animal ears. Following 4 days, the blastema ring which was created in the periphery of primary hole in the ears was removed and cultivated. The cells migrated from the blastema were expanded through 3 successive subcultures and characterized in terms of their potential differentiation, growth characteristics, and culture requirements.

***Results:*** The primary cultures tended to be morphologically heterogeneous having spindly-shaped fibroblast-like cells as well as flattened cells. Fibroblast-like cells survived and dominated the cultures. These cells tended to have the osteogenic, chondrogenic, and adipogenic differentiation potentials. They were highly colonogenic and maximum proliferation was achieved when the cells were plated at density of 100 cells/cm2 in a medium which contained 10% fetal bovine serum (FBS).

***Conclusion:*** Taken together, blastema tissue-derived stem cells from rabbit ear are of mesenchymal stem cell-like population. Studies similar to this will assist scientist better understanding the nature of blastema tissue formed at rabbit ear to regenerate the wound.

## Introduction

Throughout evolution, mammals have increasingly lost their cababilities to regenerate structures. There are limited examples of where regeneration occurs in mammals (1). One example is a scar-free regeneration of ear wound in some mammals. Rabbit was one of the first mammals discovered to have the capacity to regenerate novel tissue for repairing through-and-through holes to their ears (2). It should be mentioned that regeneration differs from repair. Unlike repair, regeneration leads to the complete replacement of the injured structures resulting in a comprehensive restoration of tissue function.

Regeneration of rabbit ear occurs by formation of a new tissue from blastema produced on the periphery of the wound. This has been investigated through experiments in which a full thickness hole was created at the animal pinna (3). The circular blastema forms around the edges of the hole and differentiate into a new sheet of cartilage in a centripetal direction. By this means, the rabbit pinna that histologically is made up of a single plate of elastic cartilage and a perichondrial layer is regenerated. Rabbit pinna is considered an effective experimental model in regeneration (4).

Regeneration of the rabbit ear is of particular interest since it shares certain aspects of the epimorphic or blastema-based regeneration occurring in vertebrate appendages. Epimorphosis is defined as a repair that occurs through dedifferentiation of the mature cells into cells with embryonic characteristics at injured site (5-6). Joseph and Dyson in 1966 described the events occurred in regeneration process in blastema formed at rabbit ear wound. According to their observation, following creating a hole in rabbit ear, epithelial cells migrate from the cut edge of the wound and form an epithelial sheet much thicker than the adjacent epidermis. They have reported that there is no derm-like tissue in the epithelial outgrowth. A few days following the injury, the thickened epithelium covers a large blastema of fibroblastic cells histologically similar to granulation tissue extending out from the edge of cartilage. In this cell aggregation, new cartilage is formed by 4 weeks and the new epidermis covers both surfaces of the holes (3). Goss *et al* have investigated the role of tissue interactions in the regeneration process in rabbit ear and found that replacement of the cartilaginous sheet requires the proximity of the healing wound to the overlying ear skin. Interestingly, they have found that skin from somewhere else of the body cannot support the regeneration (7). Patsy *et al* have been reported that the ear regeneration in rabbits is greater in male than in female animals and in pregnant than non-pregnant animals (8). 

In recent years, the study by Mahdavi Shahri *et al* is a remarkable study in that they have investigated the ultrastructure of the blastema tissue in rabbit ear during the regeneration process. According to their findings blastema tissue is a group of undifferentiated cells that are able to divide and differentiate into some parts of the body. Furthermore, they have reported that there is chondroblastic as well as endothelial cells in blastema tissue during the regeneration process (9). Mahmoudi *et al* have cultivated the undifferentiated cells from regenerating blastema of rabbit pinna and investigated them in terms of their proliferative capacity as well as the expression of particular stem cell markers. According to their findings, the cells were rather immortal cells expressing Oct4 and Sox2 stemness markers (10). 

In the current study, we attempted to further investigate the blastema tissue-derived stem cells regarding their *in vitro* differentiation potential into bone, cartilage, and adipose cells. Furthermore, we reported an optimal culture condition favoring the cell proliferation. 

## Materials and Methods

*Animal*

In this study, the use of the five New Zealand white male rabbits, almost 3-6 month old and 2.5 kg of weight was approved by the ethics committee of the Royan Institute. The animals were kept under conventional conditions. 

*Creating a through and through hole in rabbit pinna*

Blastema tissue was cultured according to the method of earlier reports with some modifications (9-10). Dorsal and ventral surface of rabbit ears were shaved using hair removing cream and anesthetized using lidocaine solution. Using a punching apparatus, a 4-mm hole was created in the animal ear. Four days after punching the hole, the blastema ring being produced in the periphery of the primary hole in the ears was removed and placed in phosphate buffer saline (PBS) and taken to the cell culture lab at Royan Institute.

*Blastema culture*

In brief, the blastema was washed twice in PBS, then immediately placed in cell culture flask containing dullbecco’s modified eagle medium (DMEM, Gibco, Germany) supplemented with 10% fetal bovine serum (FBS, Gibco, Germany) and 100 IU/ml penicillin, and 100 µg/ml streptomycin ( both from sigma, Germany) and incubated at the atmosphere of 37ºC and 5% CO2. Approximately 9-12 days following to incubation, some cells appeared to be adhering on the culture surfaces. These cells were heterogeneous in morphology and including flattened and fibroblastic cells. The culture was expanded through several subcultures. 

*Multilineage differentiation*

*Osteogenesis*

Passaged-3 cells were plated at a 6-well culture plate in DMEM containing 10% FBS and allowed to be confluent. The proliferation medium was replaced by induction medium including DMEM supplemented with 50 µg/ml ascorbic2-phosphate (Sigma, USA), 10 nM dexametazone (Sigma, USA), and 10 mM ßglycerole phosphate (Sigma, USA). Differentiation culture was maintained 21 days during which the cultures were fed twice weekly. Alizarin red staining as well as RT-PCR analysis was used to examine the differentiation.

*Adipogenesis*

Passaged-3 cells were cultivated on a 6-well culture plate and allowed to become confluent. The medium was then substituted with differentiation medium which was DMED supplemented with 100 nM dexametasone (Sigma, USA) and 50 µg/ml indometacin (Sigma, USA). Subsequently to 21 days incubation with medium replacement twice weekly, the differentiation was evaluated with oil red staining as well as RT-PCR analysis.

*Chondrogenesis*

Micro mass culture technique was used to promote blastama cell chondrogenic differentiation. Almost 2×10^5^ passaged-3 cells were suspended in a 5-ml DMEM medium in 15-ml tubes and centrifuged at 1200 rpm for 5 min. Supernatant was removed and chondrogenic medium was added on the cell pellet. This medium was DMED supplemented with 3 ng TGF beta (Transforming growth factor beta, Sigma, USA), 10 ng BMP-6 (bone morphogenetic protein-6), 50 mg/ml Transferrin-Selenium-Insulin (Sigma, USA), 50 mg Bovine serum albumin (Gibco, Germany), and 1% FBS (Gibco, Germany). The culture was maintained at 37ºC and 5% CO_2_ for 21 days afterward which the culture was prepared for light microscopy. Subsequently, five micrometer thick section was made and stained with toluidin blue. Moreover, the culture was analyzed with RT-PCR for expression of cartilage specific genes. 

**Table 1:** Primers used in RT-PCR

Target gene		Primers	Annealing temperature
	Osteocalcin	Forward primer : 5’ CTCAGCCTTCGTGTCCAA 3’ Reverse primer :5’ CTCGCACACCTCCCTCTTG 3’	57oC
	Osteopontin	Forward primer : 5’ GGCTAAACCCTGACCCATCT 3’ Reverse primer : 5’ GTGGTCATCGTCCTCATCCT 3’	59oC
	ALP	Forward primer : 5’ ACTTTGTCTGGAACCGCACT 3’ Reverse primer : 5’ GTGGTCAATCCTGCCTCCT 3’	58oC
	Sox9	Forward primer : 5’ AAGATGACCGACGAGCAG 3’ Reverse primer : 5’ GGCTTGTTCTTGCTGGAG 3’	56oC
	Aggrecan	Forward primer : 5’GGAGGTCGTGGTGAAAGGTG3’ Reverse primer : 5’CTCACCCTCCATCTCCTCTG3’	60oC
	Adiponectin	Forward primer : 5’ CGGTGAGAAGGGTGAAAAAG 3’ Reverse primer : 5’GCTGAGCGGTAGACATAG 3’	57oC
	LPL	Forward primer : 5’ TTCAACCACAGCAGCAAGAC 3’ Reverse primer : 5’ TAACAGCCAGTCCACCACAA 3’	57oC
HKG	GAPDH	Forward primer: 5’ gga gaa acc tgc caa gta tg 3’ Reverse primer : 5’ tga gtg tcg ctg ttg aa gtc 3’	60oC

*RT-PCR analysis*

Total RNA was isolated from the differentiated cells using the RNX^TM ^(-Plus) (CinnaGen Inc., Tehran, Iran). In order to eliminate residual DNA, the RNA was treated with 1 U/ml of RNase-free DNase I (Fermentas, Opelstrasse 9, Germany) per 1 mg of RNA in the presence of 40 U/ml of ribonuclease inhibitor (Fermentasm, Germany) and 1×reaction buffer with MgCl_2_ for 30 min at 37°C. Standard RT reactions were then performed with 2 μg total RNA using oligo (dt) as a primer and a RevertAidTM First Strand cDNA Synthesis Kit (K1622, Fermentas, Germany) according to the manufacturer’s instructions. For every reaction set, one RNA sample was prepared without RevertAidTMM-MuLV Reverse Transcriptase (RT- reaction) to provide a negative control in the subsequent PCR. To minimize variation in the RT reaction, all RNA samples from a single experimental setup were reverse transcribed simultaneously. Reaction mixtures for PCR included 2 ml cDNA, 1×PCR buffer (AMSTM, CinnaGen Co., Tehran, Iran), 200 mM dNTPs, 0.5 mM of each antisense and sense primer (Table 1), and 1 U Taq DNA polymerase. 

*Culture optimization*

In general, cell growth is dependent on the presence of serum and initial seeding density at culture initiation. In order to determine the best serum concentration as well as the best cell seeding density, passaged-3 isolated cells were cultivated at varying density of 100, 500, 1000, 2000, 5000, and 10000 cells/cm^2^ and in the presence of different FBS concentration including 5%, 10%, and 15%, respectively. One week after plating the cells, the cultures were appraised using MTT assay for viable cells. The experiments were replicated three times. The average value of obtained data was analyzed using one-way ANOVA.

*MTT assay*

In order to determine the viable cell number in the culture, MTT [3-(A, 5-dimethylthiazol-2-yl)-1, 5-diphenyl tetrazulium bromide] (MTT, Sigma, USA) assay was performed. The cells were washed with PBS, provided with MTT (5 mg/ml in PBS) solution diluted in DMEM in 1:5 ratios and incubated for 2 hr at 37 ºC. The MTT was then replaced by 0.5 ml of extraction solution (dimethylsulphoxide: DMSO). The absorbance of the supernatant was then recorded using a microplate reader (BioTek EL x800, USA) at 540 nm. Cell number was determined through a standard curve that was established by using a known cell number. 

*Growth curve*

In order to plot growth curve, cell culture must be observed in daily-basis to determine the cell number. Therefore, blastema tissue-derived stem cells from passage 3 were cultivated at 10^4^ per/well in 24-well culture plates and allowed to proliferate for a period of 2-weeks. Some wells were then trypsinized per days and the cells were counted using hemocytometer. Using the data, a growth curve was finally plotted.

*Colonogenic activity*

In order to examine the blastema tissue-derived stem cells colonogenic activity, colony forming unit-fibroblast (CFU-F) assay was performed. About 100 cells from passaged-3 culture were plated in 60 mm dish and allowed to form colony for a period of 7 days. At the end of this period, the culture was fixed with 10% formalin and stained with crystal violet for 5 min. The number of colonies was then counted under phase contrast invert microscope. Furthermore, the size of colonies was measured using a microscopic objective micrometer. CFU-F assay was replicated 5 times.

## Results

*Blastema culture*

**Figure 1:**
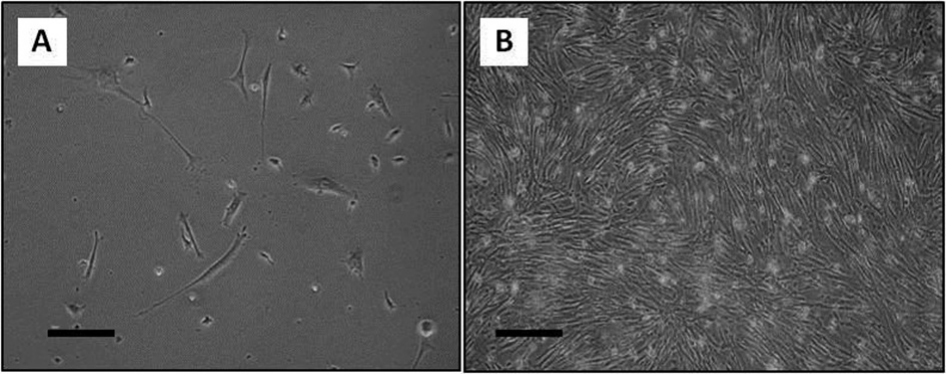
Blastema cell culture. A) At primary culture, varying cell morphologies including elongated as well as flattened cells had been presented. B) Fibroblastic cells dominated the cultures at following subcultures (Bar= 100 µm

Cell culture was observed daily with a phase contrast invert microscope. According to the observations, about 9-12 days following to culture initiation, some cells tended to migrate from the blastema ring and became the origin of culture. These cells were adherent. The culture was first heterogeneous in term of cell morphology (Figure 1A). Only fibroblast-like cells formed small colonies which then grew and became confluent (Figure 1B). Following a few subcultures, fibroblastic cells dominated the culture. These cells were propagated with a few subcultures and used at the rest of experiment as blastema tissue-derived stem cells.

*Osteogenesis*

**Figure 2:**
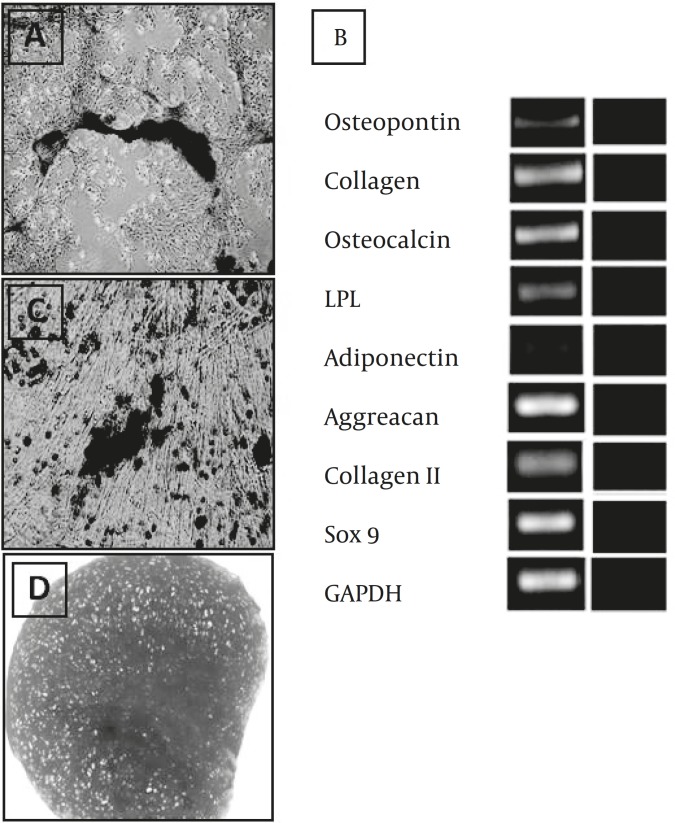
Multilineage differentiation potential of the blastema tissue-derived stem cells from rabbit ear. A) Osteogenic culture stained with alizarin red. B) Adipogenic culture stained with oil red. C) Chondrogenic culture stained with toluidine blue. D) Lineage specific genes expressed in the differentiated cultures (Bar= 100 µm

This culture was observed daily using an invert microscope. The early signs of morphologic changes and osteogenic differentiation occurred a week following to induction. In some area of monolayer cultures, mineralized aggregates were formed. These osteogenic foci were increased as the time advanced. Three weeks after induction initiation, the cultures turned into red upon alizarin red staining (Figure 2A). According to RT-OCR analysis at osteogenic culture, bone specific genes including osteocalcin, osteopontin, and ALP were expressed (Figure 2B).

*Adipogenic culture*

According to our observations, the first lipid droplet was appeared on day 5 of culture in some cells. These droplets developed in other cells on the following days therefore at the end of differentiation period most of the cells contained lipid droplets. The droplets were positively stained red with Oil red staining (Figure 2C). RT-PCR analysis indicated that the adipose-specific genes including adiponectin and LPL were expressed in the differentiated cells (Figure 2B). 

*Chondrogenic differentiation*

The sections prepared from the chondrogenic cultures indicated metachromasia following the toluidin blue staining (Figure 2D). According to RT-PCR analysis, the mRNA of cartilage specific genes including aggreacan, and Sox9 were produced in the differentiated cells (Figure 2B).

*Culture optimization for blastema cell culture*

**Figure 3:**
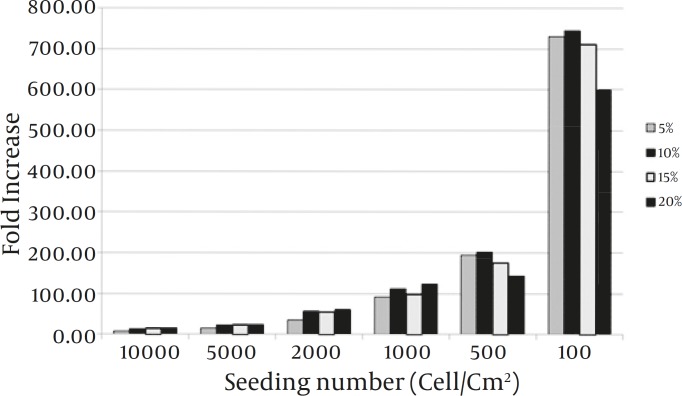
Culture optimization for increased proliferation of the blastema tissue-derived stem cells from rabbit ear. The cells appeared to have a higher proliferation when cultivated at 100 cells in the presence of a medium containing 10% FBS. In this regard, the difference between 10% and 15% and also 10% and 5% was not significant

The maximum increase in cell yield was achieved when the cells cultivated at density of 100 cells/cm^2^ in a medium containing 10% FCS (Figure 3).

*Growth curve*

**Figure 4:**
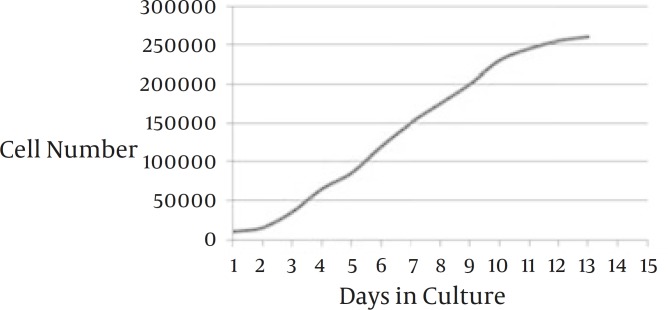
Growth curve plotted for blastema tissue-derived stem cells from the rabbit ear. The cells tended to have a short period of lag phase and a prominent log phase. The culture reached to plateau at day 10

Monitoring daily growth of blastema tissue-derived stem cells during 14 day-culture period indicated that the rate of cell proliferation was considerable. Lag phase was about one day following which the cells started to enter into log phase when a relatively rapid proliferation occurred. At day 10 the plateau phase was reached (Figure 4). 

*Colonogenic assays*

**Figure 5:**
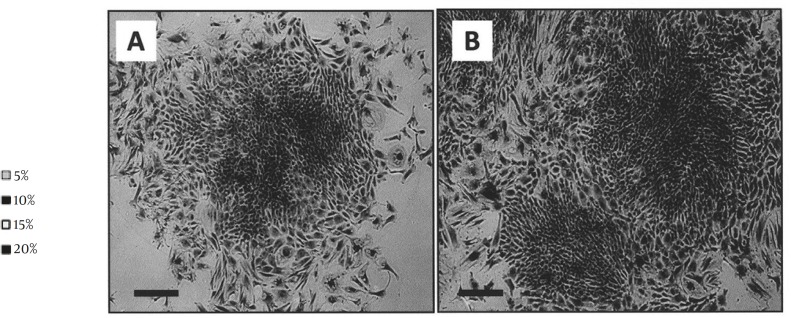
Crystal violet staining of the colonies from rabbit blastema cell culture. A) Blastema tissue-derived stem cells tended to be highly colonogenic producing large colonies at low density culture. B) The colonies became confluent resulting in formation of a monolayer cells (Bar= 100 µm

The colonies produced at blastema cell culture stained purple following crystal violet staining. According to CFU-F assay, blastema tissue-derived stem cells appeared as colonogenic cells producing average of 77.72±8.65 colonies within average dimension of 1.21±0.37 mm^2^ per each 100 cells plated in the culture dish (Figure 5).

## Discussion

In the present study, progenitor cells from blastema tissue of rabbit ear were expanded and characterized in terms of their *in vitro* differentiation capacity into some skeletal cell lineages. It has been formerly suggested that the blastema from rabbit ear is consisted of a group of undifferentiated cells that are able to divide and differentiate along chondrocytic cell lineages *in vivo *in order to regenerate ear wound (9). Moreover, the population of stem cells from blastema tissue has already been cultivated and described as immortal cells expressing Oct4 and Sox2 markers (10). There was no information regarding *in vitro* differentiation capacity of blastema tissue-derived stem cells. A study like this may assist scientists better understand blastema nature and the regeneration capacity that is restricted to specific locations in some mammalians including rabbit as well as mice of the MRL strain (11). In most mammals including human, the lost tissue especially in cartilage is replaced by a scar rather than a functionally active original tissue. 

We found that blastema tissue-derived stem cells were able to differentiate along bone, cartilage, and adipose cells, a property that is characteristic of mesenchymal stem cells (MSCs). Moreover, according to our findings blastema tissue-derived stem cells tended to propagate in colonogenic manner *in vitro*. Colony formation is the other feature described for MSCs (12-14). MSCs are adult stem cells first isolated from bone marrow (15-16) and then reported as the resident of many other tissues including adipose tissue, cord blood, amniotic fluid, peripheral blood, bone, cartilage, and muscle tissues (17-23). According to the findings by the present study, it seems that blastema tissue-derived stem cells from rabbit ear are of the MSC population. 

Blastema tissue formed at rabbit punched ear consisted of a mass of central dedifferentiated cells originated from the ear cartilage and a peripheral epithelium grown from the adjacent epidermis (3). In order to isolate the progenitor cells from the blastema tissue, in the present study, the blastema ring obtained from punched ear was cultured as an explant. The cells that migrated from the blastema and became the origin of culture may have originated from either the central cells or the covering epithelium. According to our observations, in the early days of the primary culture, morphologically distinct cell populations were present such as fibroblast-like cells (probably from blastema central region) as well as flattened cells (from the covering epithelium). Based on our findings, only the fibroblastic cells survived in the subsequent subcultures. Therefore, it could be speculated that the progenitor cells isolated from the blastema could be coming from the blastema central cells. 

Progenitor cells from the blastema have already been reported as highly proliferative cells. According to Mahmoudi *et al* experimental works, these cells are rather immortal cells capable of growing for more than 120 passages in culture (10). Our findings are also in agreement with this data. According to the growth curve plotted for the cells, the blastema progenitor cells tended to rapidly divide and reach plateau. Furthermore, we noticed that, at the plotted curve, the lag phase was very short (about one day) indicating the rapid adaptation of the studied cells with culture conditions. This is in line with earlier investigations which suggested that stem cells are resistant to culture stressful conditions (24). 

Moreover, the best FBS concentration for efficient proliferation of the stem cells from blastema tissue appeared to be 10%. Mahmoudi *et al* also cultivated the cells in presence of 5, 10, and 15% FBS and found that 15% FBS containing medium is associated with more cell proliferation (10). It should be mentioned that in our study, the difference between 10% and 15% was not significant. This difference, however, may arise from the different cell density that is used to initiate the culture. We established the cultures with 100, 500, 1000, 2000, 5000, and 10000 cells/cm^2^ in the presence of varying FBS concentrations and found that when the culture was initiated by 100 cells/cm^2^ in a medium containing 10% FBS, the maximum increase in cell yield could be achieved. In the study by Mahmoudi *et al* cell density value had not been revealed. 

Furthermore, fibroblast-like cells derived from the regenerating blastema had the capability to differentiate along three skeletal cell lineages of bone, cartilage, and adipose cells. The chondrogenic capacity of the cells is more understandable than their osteogenic and adipogenic potential since the studied cells have been isolated from the repair tissue formed at cartilage defect which was experimentally created in rabbit pinna. There would be a logical explanation for their bone and adipose differentiation. One possibility would be that the cells themselves had no proper differentiation potential but the culture condition provided for them (i.e., osteogenic and adipogenic inducers present in differentiation medium) forced the cells to adopt corresponding differentiated phenotypes. The other possibility is that the cells possessed a true tripotent differentiation capacity. 

## Conclusion

Taken together, a fibroblast-like cell population was isolated from the blastema tissue being formed at punched rabbit ears. Our evaluation indicated that the cells had similar characteristics as MSCs: ability to differentiate into three cell lineages including bone, cartilage, and adipose cells and the capacity to form a colony. Studies similar to this could assist scientists to better understand the nature of blastema tissue that is responsible for regeneration occurring at a tissue defect created in rabbit ear. 
